# Relationship Between Health and Well-Being and Adherence to Social Norms Across Adulthood

**DOI:** 10.1093/geroni/igaa057.1000

**Published:** 2020-12-16

**Authors:** Giselle Ferguson, Martin Sliwinski, Stacey Scott

**Affiliations:** 1 Stony Brook University, Stony Brook, New York, United States; 2 Penn State University, University Park, Pennsylvania, United States

## Abstract

When an individual deviates from social norms, others implement stressful social consequences to discourage deviance. Over a lifetime, this stress may accumulate, causing physiological wear-and-tear and poorer health and well-being. However, older adults may choose to surround themselves primarily with close, supportive others, so they may no longer be exposed to consequences for deviance, and would then be shielded from downstream effects. Alternatively, adults from older cohorts may value traditional norms more strongly, and would therefore experience greater effects of norm deviance. We expected that individuals who adhered to norms would report better health and well-being than individuals who deviated from norms, and that this effect would be either absent or intensified for older adults. We tested this prediction with a secondary data analysis of n=220 adults ages 25-65 (63.2% women). We operationalized individuals’ adherence to their respective gender norms and to general, non-gendered norms using demographic and questionnaire responses. Greater general norm adherence significantly predicted better health and well-being, independent of age. For masculine norms, work-primacy norm adherence predicted better well-being, but anger and self-reliance norm adherence predicted worse health and well-being in men. Age moderated this effect: with older age, the effect of anger was reversed such that greater anger predicted better health among men. For feminine norms, adherence did not predict better outcomes in women at any age. Although the relationship between gendered norm adherence and health and well-being may depend on cohort, the results suggest that general norm adherence does influence health and well-being.

